# Sex-Specific Differences in Mortality and Incident Dialysis in the Chronic Kidney Disease Outcomes and Practice Patterns Study

**DOI:** 10.1016/j.ekir.2021.11.018

**Published:** 2021-12-01

**Authors:** Manfred Hecking, Charlotte Tu, Jarcy Zee, Brian Bieber, Sebastian Hödlmoser, Helmut Reichel, Ricardo Sesso, Friedrich K. Port, Bruce M. Robinson, Juan Jesus Carrero, Allison Tong, Christian Combe, Bénédicte Stengel, Roberto Pecoits-Filho

**Affiliations:** 1 Clinical Division of Nephrology and Dialysis, Department of Internal Medicine III, Medical University of Vienna, Vienna, Austria; 2 Arbor Research Collaborative for Health, Ann Arbor, Michigan, USA; 3 Department of Epidemiology, Center for Public Health, Medical University of Vienna, Vienna, Austria; 4 Nephrologisches Zentrum Villingen-Schwenningen, Villingen-Schwenningen, Germany; 5 Nephrology Division, School of Medicine, Universidade Federal de São Paulo, São Paulo, Brazil; 6 Department of Medical Epidemiology and Biostatistics, Karolinska Institutet, Stockholm, Sweden; 7 Centre for Kidney Research, The Children’s Hospital at Westmead, Westmead, Australia; 8 Sydney School of Public Health, The University of Sydney, Camperdown, Australia; 9 Service de Néphrologie Transplantation Dialyse, Centre Hospitalier Universitaire de Bordeaux, Bordeaux, France; 10 Université Paris-Saclay, UVSQ, Institut National de la Santé et de la Recherche Médicale, Clinical Epidemiology Team, CESP (Research Center in Epidemiology and Population Health), Villejuif, France; 11 School of Medicine, Pontificia Universidade Catolica do Parana, Curitiba, Brazil

**Keywords:** chronic kidney disease, competing risks, gender disparity, kidney replacement therapy, mortality, sex-specific differences

## Abstract

**Introduction:**

More men than women start kidney replacement therapy (KRT) although the prevalence of chronic kidney disease (CKD) is higher in women than men. We therefore aimed at analyzing sex-specific differences in clinical outcomes among 8237 individuals with CKD in stages 3 to 5 from Brazil, France, Germany, and the United States participating in the Chronic Kidney Disease Outcomes and Practice Patterns Study (CKDopps).

**Methods:**

Fine and Gray models, evaluating the effect of sex on time to events, were adjusted for age, Black race (model A); plus diabetes, cardiovascular disease, albuminuria (model B); plus estimated glomerular filtration rate (eGFR) slope during the first 12 months after enrollment and first eGFR after enrollment (model C).

**Results:**

There were more men than women at baseline (58% vs. 42%), men were younger than women, and men had higher eGFR (28.9 ± 11.5 vs. 27.0 ± 10.8 ml/min per 1.73 m^2^). Over a median follow-up of 2.7 and 2.5 years for men and women, respectively, the crude dialysis initiation and pre-emptive transplantation rates were higher in men whereas that of pre-KRT death was more similar. The adjusted subdistribution hazard ratios (SHRs) between men versus women for dialysis were 1.51 (1.27–1.80) (model A), 1.32 (1.10–1.59) (model B), and 1.50 (1.25–1.80) (model C); for pre-KRT death, were 1.25 (1.02–1.54) (model A), 1.14 (0.92–1.40) (model B), and 1.15 (0.93–1.42) (model C); for transplantation, were 1.31 (0.73–2.36) (model A), 1.44 (0.76–2.74) (model B), and 1.53 (0.79–2.94) (model C).

**Conclusion:**

Men had a higher probability of commencing dialysis before death, unexplained by CKD progression alone. Although the causal mechanisms are uncertain, this finding helps interpret the preponderance of men in the dialysis population.

## Graphical abstract

**Figure:**
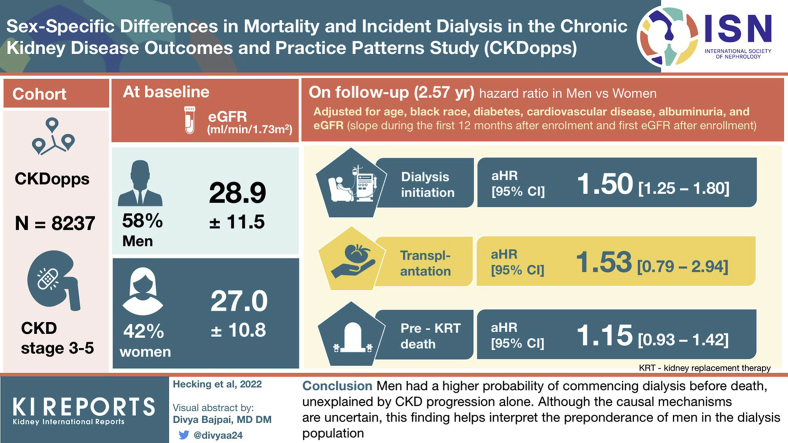

See Commentary on Page 375

More than 850 million population are expected to suffer from CKD in 2021.^1^ Most of these patients with CKD are women.^2^, ^3^, ^4^ Surprisingly, many analyses, such as from the international Dialysis Outcomes and Practice Patterns Study,^5^ have revealed that among individuals receiving therapy for kidney failure (prevalent patients on hemodialysis), women clearly constitute the minority, at approximately 40% (vs. 60% men). Unequal sex distribution at the start of dialysis (dialysis incidence) is the reason for the higher prevalence of men on dialysis, as the male-to-female proportions were >50% (men) to <50% (women) among those who initiated dialysis in >30 countries.^6^ This rate has remained historically constant in the last 50 years.^7^^,^^8^

The discrepancy between more women than men having CKD on the one hand, but more men than women receiving KRT on the other hand, has been known for decades.^9^ Nevertheless, this finding has not been convincingly explained, despite growing interest in studying sex differences in medicine in general,^10^, ^11^, ^12^ and in kidney disease in particular.^13^ Some researchers have suggested that biological reasons exist for the preponderance of men on KRT. Among biological reasons, progression of kidney disease has been the most debated,^2^ but a recent systematic review arrived at the conclusion that the decline of kidney function does occur faster in men than in women.^4^

Nonbiological reasons for the sex differences between CKD and KRT are more difficult to verbalize. On the basis of meaningful country differences in the percentages of women receiving dialysis in the Dialysis Outcomes and Practice Patterns Study, we have previously speculated that disparities between men and women might explain an under-representation of women relative to men on KRT.^5^ The term disparity in this context portends the connotation that the observed differences might be unfair toward women. For example, women with CKD in the United States report more frequently than men that they have “never been told by a doctor or other health professional that they had weak or failing kidneys.”^14^^,^^15^ Moreover, the hypothesis that women more often than men lack a caregiver has been brought forth as an explanation for the finding that elderly women with late-stage CKD more often than men choose palliative care over dialysis.^16^^,^^17^ A more refined explanation than gender disparity has been provided by a recent review on “sex and gender as modifiers of health, disease and medicine” (in general).^18^ Therein, the authors acknowledge that gender constructs determine “individual use of the health care system and that being perceived as a man or a woman triggers different responses from clinicians who might diagnose and suggest interventions differently according to gender” (gender here being different from sex, which is biological).

Individuals diagnosed with having CKD who have become aware of their condition^19^ should prompt initiation of nephrology care in a specialized CKD facility. Nevertheless, as CKD (in contrast to acute kidney injury) is not generally reversible, the typical journey of a patient with CKD in the clinic will at some point encompass KRT initiation, by dialysis or transplantation. Competing with the probability (the “chance” or need) to initiate dialysis is the risk of dying in the predialysis (pre-KRT) stage. In the present analysis, we asked whether the risk of dying among women relative to their chance of initiating *KRT* is similarly proportioned as in men within the CKD population under nephrological care. Aiming to evaluate these and other clinical outcomes by sex, including to determine differences in treatment and patient characteristics between men and women, we analyzed data from the CKDopps, which systematically captures information from real-world, nephrologist-run CKD clinics. To provide a meaningful addition on top of 3 previous analyses, from the United States,^20^ Italy,^21^ and Sweden,^22^ we emphasized country-specific comparisons. One of the key tasks, before the analysis and sex-specific comparison of outcomes, was to describe the sex-specific prevalence of CKDopps participants at baseline, which was presumed to be a function of patient referral from the general population onto specialized CKD care.

## Methods

### Patients and Data Collection in CKDopps

Initiated in 2013, CKDopps is an ongoing, international prospective cohort study that recruits nondialysis-dependent individuals aged >18 years with CKD in stage 3 and above (eGFR <60 ml/min per 1.73 m^2^). The study design of CKDopps has been published previously.^23^ Briefly, CKDopps participants are sequentially selected from samples of nephrologist-run CKD clinics^24^ until the target number of patients per clinic (60–80 patients) is reached. The study adheres to the Declaration of Helsinki and has received institutional review board approval in the United States and as required by the national authorities of the participating countries. All CKDopps patients provided written informed consent before enrollment.

Patient demographics and comorbidities are captured at study enrollment. Routine laboratory data, medication prescriptions, and KRT preparation data (vascular access creation and enrollment on the transplant waiting list) are captured at study enrollment and longitudinally, up to a monthly frequency based on how often these data are measured for clinical care. KRT (including transplant and start of dialysis), hospitalization, and mortality events are captured continuously during study follow-up. In Brazil, France, and the United States, clinical data are captured by medical record abstraction, whereas in Germany, data are captured from electronic health records after data review performed by a study coordinator to evaluate data quality.^24^

### Derivation of the Study Cohort

**Figure 1:**
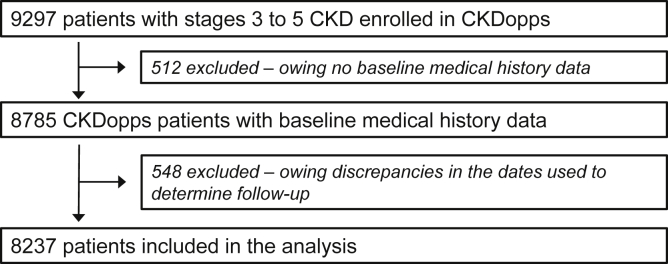
Consort diagram of study. CKD, chronic kidney disease; CKDopps, Chronic Kidney Disease Outcomes and Practice Patterns Study.

For the present analyses, we included patients from Brazil, France, Germany, and the United States. In these CKDopps countries, 9297 patients (from 136 nephrology clinics), specifically 1012 (18) from Brazil, 2969 (41) from France, 2772 (33) from Germany, and 2544 (44) from the United States, had been enrolled as of 2013 to 2019. There were 512 patients who had to be excluded because baseline demographics and medical history were missing. In addition, 548 patients had to be further excluded from the current analyses owing to discrepancies in the dates used to determine follow-up, yielding a final cohort of 8237 patients for the present analysis (Figure 1). There were no additional exclusions owing to patient death or loss of follow-up during the lead-in period.

### Recording of the Patient Sex

Recording of patient sex in CKDopps occurs in the form of a binary variable (male vs. female), marked on the questionnaire by the country-specific CKDopps investigator filling out the form, without differentiating between sex (male vs. female) and gender (man vs. woman) or transgender.^25^ Throughout the current manuscript, CKDopps participants of male and female sexes are referred to as men and women, respectively, to remain consistent with previous work.^5^^,^^7^

### Data Analysis

Descriptive statistics were used to describe baseline demographic and clinical characteristics of patients by country and sex. eGFR was calculated using the CKD Epidemiology Collaboration formula.^26^ CKD stage was defined using the following eGFR cut-points: stage 3 (eGFR 30–59 ml/min per 1.73 m^2^), stage 4 (eGFR 15–29 ml/min per 1.73 m^2^), and stage 5 (eGFR <15 ml/min per 1.73 m^2^). A linear mixed regression model with random intercepts and random slopes was used to estimate individual eGFR change over follow-up time; patients with at least 1 postbaseline eGFR measurement were included in the model. The closest eGFR within 6 months before KRT initiation was reported as the eGFR at KRT initiation. The most recent KRT preparation data (vascular access creation and enrollment on the transplant waiting list) within 1 year before the patient’s first eGFR <20 ml/min per 1.73 m^2^ were included.

The maximum of follow-up time among patients in Brazil was 3.35 years, so all outcome-related analyses were restricted to 3 years of follow-up. The cumulative incidences of (i) hemodialysis or peritoneal dialysis, (ii) kidney transplantation, and (iii) death before the occurrence of either of the 2 KRT practices were estimated by CKD stage at study entry and sex. Fine and Gray^27^ proportional hazards models accounting for competing risks were used to evaluate the associations between sex and each event with adjustment for different combination sets of covariates, including age, Black race, diabetes comorbidity, cardiovascular disease, albuminuria, and individual eGFR slope during the first 12 months of follow-up. The eGFR slope during this 12-month lead-in period was calculated using a linear mixed model accounting for repeated measures within a patient with random intercepts and random slopes. Time at risk started 12 months after study enrollment until the occurrence of an event or censoring at end of patient follow-up (owing to clinic transfer, loss to follow-up, or up to 3 years of follow-up). The analyses excluded 544 who reached KRT before 12 months, 314 who died before 12 months, and 901 who departed for other reasons.

Missing data for all independent variables in subdistribution hazards models were multiply imputed by chained equations, and results from 20 imputed data sets were combined for the final analysis using Rubin’s formula. All statistical analyses were conducted using SAS, version 9.4 (SAS Institute Inc., Cary, NC). This study was supported by a grant from the Austrian Science Fund (FWF grant number KL754-B).

## Results

### Sex-Specific Differences in Characteristics of the Study Cohort at Baseline

More men than women were enrolled in the CKDopps in each of the 4 countries, presumably reflecting the underlying sex distribution in the participating nephrologist-run clinics (Table 1). Sex-specific CKD prevalence has previously been reported for France, Germany, and the United States,^2^ but for more direct comparison with the CKDopps data set (at least in the United States), we used data from the National Health and Nutrition Examination Survey (2013–2018) to calculate the percentage of women among all individuals with CKD, by CKD stage and age group (Supplementary Table S1). We observed that the discrepancy between women with CKD (National Health and Nutrition Examination Survey) versus women who were actually treated for CKD in nephrology clinics (CKDopps) increased with older age (in the United States where these data are directly comparable, but also in the other CKDopps countries).

**Table 1:** Patient characteristics at baseline by country and sex

Parameter	All		Brazil		France		Germany		US	
	Men	Women	Men	Women	Men	Women	Men	Women	Men	Women
Patients, *n*	4811	3426	398	366	1944	1024	1480	1110	989	926
Demographics										
Age, yr	71 [63–78]	71 [61–79]	67 [58–75]	66 [55–76]	69 [62–77]	67 [58–76]	74 [66–79]	76 [69–82]	71 [62–78]	70 [61–78]
Black race, %	7	10	25	27	3	2	–	–	17	26
Marital status, %										
Married	72	47	76	51	74	52	–	–	68	42
Widowed	7	26	11	31	6	24	–	–	8	27
Divorced	9	12	4	5	10	12	–	–	10	14
Single (never married) or separated	11	14	9	14	10	12	–	–	14	17
Employed^a^, %	53	39	44	24	71	56	–	–	36	31
Smoker, %	10	6	7	5	13	10	6	2	11	7
Education < high school, %	18	26	59	62	12	20	–	–	12	16
Clinical status										
Body mass index, kg/m^2^	29.0 (5.3)	29.7 (7.0)	27.8 (5.0)	28.2 (5.5)	28.6 (5.1)	29.0 (7.1)	28.9 (5.2)	29.3 (6.2)	30.9 (6.2)	32.3 (7.8)
Clinic visit frequency, per year	2.6 [1.7–4.0]	2.9 [1.9–4.1]	4.0 [2.8–6.0]	4.0 [2.8–5.5]	2.1 [1.4–2.9]	2.0 [1.4–3.0]	–	–	3.6 [2.4–5.3]	3.7 [2.5–5.0]
Characteristics of CKD										
eGFR, ml/min per 1.73 m^2^	28.9 (11.5)	27.0 (10.8)	26.6 (11.9)	25.3 (11.5)	32.8 (11.5)	31.2 (11.0)	26.0 (10.0)	25.2 (9.2)	26.4 (11.1)	25.3 (10.6)
CKD stage										
Stage 3	39	33	35	30	56	51	23	19	33	30
Stage 4	53	58	50	52	41	44	70	76	53	55
Stage 5	8	9	15	19	4	5	7	6	14	15
CKD vintage,^b^ yr	4.6 [2.2–8.6]	4.1 [1.8–8.1]	2.7 [0.8–5.6]	2.8 [1.1–6.1]	5.2 [2.6–9.9]	5.1 [2.3–10.4]	–	–	4.1 [1.7–7.5]	3.6 [1.5–6.8]
Reported cause of CKD,^c^ %										
Diabetes	29	29	36	39	21	19	32	29	36	37
Hypertension	30	30	35	29	22	18	34	37	36	33
Glomerulonephritis/vasculitis	17	12	8	11	26	21	11	6	8	9
Tubulointerstitial disease	7	11	8	11	10	17	4	9	3	5
Polycystic	4	5	3	4	4	8	3	3	2	3
Other	12	12	6	3	10	11	15	15	12	10
Unknown	1	2	3	4	6	7	1	1	3	3
Albuminuria or equivalent^d^										
Normal to mildly increased	27	38	43	47	24	34	28	44	31	37
Moderately increased	28	28	21	19	31	32	29	32	22	23
Severely increased	45	33	36	34	45	34	44	24	47	41
Comorbidities, %										
Coronary artery disease	33	21	25	20	30	15	35	22	37	26
Cerebrovascular disease	12	9	12	7	14	7	10	10	11	10
Congestive heart failure	15	14	16	15	14	11	14	13	17	18
Other cardiovascular disease	25	20	14	13	29	24	24	19	24	20
Peripheral vascular disease	21	14	24	23	20	12	22	14	18	14
Hypertension	89	89	91	91	91	90	84	85	92	93
Diabetes	47	45	49	45	46	39	45	41	54	56
Cancer (nonskin)	20	13	12	5	24	17	17	11	20	16
Gastrointestinal bleeding	1	1	2	3	1	1	2	2	2	1
HIV/AIDS	0.6	0.4	1.2	0.8	1	1	0.1	0	1.2	0.3
Lung disease	10	9	9	6	11	9	8	6	13	13
Neurologic disease	3	4	11	13	2	3	2	2	4	6
Any psychiatric disorder^e^	7	13	11	21	7	14	4	6	12	19
Depression	5	12	8	21	5	13	2	4	9	17
Recurrent cellulitis/gangrene	3	3	7	6	–	–	1	2	4	4
Laboratory^f^										
S. phosphorus, mg/dl	3.7 (1.5)	3.9 (0.8)	4.1 (1.0)	4.4 (1.2)	3.5 (2.0)	3.8 (0.7)	3.6 (0.8)	3.8 (0.7)	3.9 (1.1)	3.9 (0.9)
S. calcium, mg/dl	9.4 (5.6)	9.4 (0.7)	9.4 (0.7)	9.3 (0.8)	9.6 (8.3)	9.5 (0.6)	9.2 (0.6)	9.4 (0.7)	9.1 (0.6)	9.3 (0.6)
S. PTH, pg/ml	92 [54–151]	92 [57–157]	97 [53–188]	97 [62–188]	79 [49–130]	84 [54–138]	111 [68–187]	100 [62–166]	103 [62–165]	100 [62–180]
S. potassium, mEq/l	4.6 (0.6)	4.5 (0.6)	4.8 (0.6)	4.8 (0.7)	4.6 (0.5)	4.5 (0.5)	4.7 (0.7)	4.5 (0.6)	4.6 (0.6)	4.5 (0.6)
S. albumin, g/dl	4.0 (0.6)	3.9 (0.6)	4.0 (0.6)	3.8 (0.7)	4.0 (0.5)	4.0 (0.5)	4.1 (0.7)	4.2 (0.6)	3.8 (0.6)	3.8 (0.5)
Hemoglobin, g/dl	12.8 (1.9)	11.9 (1.5)	12.6 (2.1)	11.6 (1.6)	13.3 (1.7)	12.3 (1.4)	12.6 (1.8)	12.0 (1.4)	12.2 (2.0)	11.3 (1.6)
Ferritin, ng/ml	143 [81–259]	120 [60–225]	175 [93–383]	136 [62–239]	138 [81–245]	109 [58–186]	145 [75–271]	125 [60–248]	159 [75–287]	144 [62–301]
TSAT, %	24 [18–30]	22 [17–29]	27 [20–34]	24 [19–33]	24 [19–30]	22 [17–29]	23 [18–29]	21 [16–27]	21 [17–30]	21 [1–25]
CRP,^g^ mg/dl	0.5 [0.2–1.2]	0.5 [0.2–1.4]	–	–	0.4 [0.2–0.8]	0.4 [0.2–0.9]	0.7 [0.3–2.0]	0.7 [0.2–2.2]	–	–
Systolic blood pressure, mm Hg	138 (21)	139 (21)	132 (19)	135 (21)	143 (20)	142 (21)	136 (21)	138 (22)	136 (20)	137 (21)
HbA1c,^h^ %	6.9 [6.3–7.7]	7.1 [6.3–8.1]	6.8 [6.2–7.8]	7.3 [6.2–8.2]	6.9 [6.3–7.6]	7.1 [6.4–8.2]	6.9 [6.4–7.8]	6.9 [6.2–7.8]	6.9 [6.3–8.1]	7.0 [6.1–8.1]
S. glucose,^h^ mg/dl										
Fasting	133 [107–163]	128 [101–166]	116 [95–158]	130 [97–191]	131 [108–157]	127 [101–157]	139 [106–187]	137 [103–175]	136 [107–187]	126 [100–185]
Nonfasting	138 [107–187]	139 [107–190]	112 [98–133]	132 [104–160]	144 [109–174]	133 [117–162]	142 [109–194]	146 [115–196]	146 [112–192]	139 [105–198]

Values reported in mean (SD) or median [interquartile range].

CKD, chronic kidney disease; CKDopps, Chronic Kidney Disease Outcomes and Practice Patterns Study; CRP, C-reactive protein; eGFR, estimated glomerular filtration rate; HbA1c, hemoglobin A1c; KDIGO, Kidney Disease: Improving Global Outcomes; N, number; S., serum; PTH, parathyroid hormone; TSAT, transferrin saturation; US, United States.

a Restricted to age <65 years old.

b Time already spent in the CKD clinic on study inclusion into CKDopps.

c 10% missing in Brazil and 35% missing in the US.

d Thresholds from KDIGO 2012 guidelines: normal or mildly increased (<30 mg/g); moderately increased (30–300 mg/g); severely increased (>300 mg/g); % of missing data is 51% in Brazil, 9% in France, 49% in Germany, and 46% in the US.

e Includes depression, bipolar disorder, schizophrenia/psychotic disorder, and alcohol or other substance abuse within past 12 months.

f The most recent value within 6 months before CKDopps enrollment.

g Results for Brazil and US are suppressed owing to high % missingness (>90%); 51% and 52% missingness in French and German data, respectively.

h Restricted to patients with diabetes.

The median age of the study cohort was 71 years, with relatively younger patients included in Brazil and relatively older patients included in Germany (Table 1). As also found in Table 1, men were more frequently married than women, which seemed to be explained by both a much lower percentage of widowed men when compared with widowed women and a lower percentage of divorced men when compared with divorced women. Men were more frequently smokers than women, and men were more frequently employed. The median (interquartile range) clinic visit frequency per year was slightly (not significantly) smaller in men (2.6 [1.7–4.0]) than in women (2.9 [1.9–4.1] for women). Among comorbidities, all vascular diseases including coronary artery disease were more frequent among men versus women. Depression was less frequent among men (5%) versus women (12%). There were no striking sex differences in laboratory parameters.

### Characteristics of CKD at Baseline and Details of KRT Initiation by Sex

The mean ± SD eGFR at baseline (an individual’s study inclusion in CKDopps) was 28.9 ± 11.5 ml/min per 1.73 m^2^ in men versus 27.0 ± 10.8 ml/min per 1.73 m^2^ in women and was higher in men versus women in all countries (Table 1). These findings were consistent with a higher fraction of patients with CKD stage 3 when compared with patients with CKD stages 4 and 5 among men in all 4 countries. Furthermore, in line with these data, the median time already spent in the CKD clinic on study inclusion in CKDopps (“vintage”) was 4.6 years in men and 4.1 years in women. The fractions of reported causes of CKD were rather similar among both sexes (some differences regarding glomerulonephritis/vasculitis and tubulointerstitial disease). Albuminuria, a marker of kidney injury, more frequently was moderately and severely increased among men.

**Figure 2:**
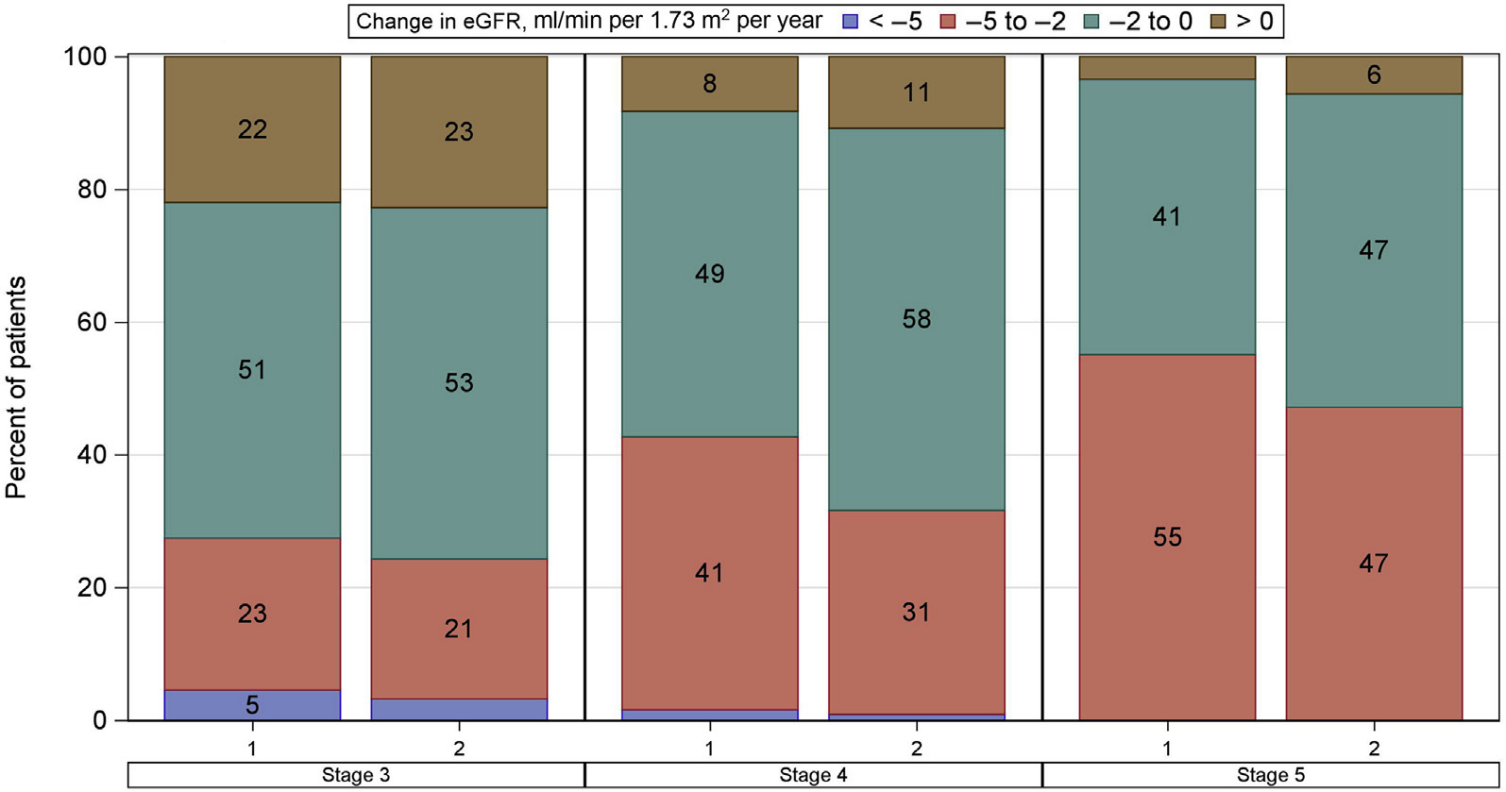
Yearly change in eGFR in the follow-up by CKD stage at study entry and sex. CKD, chronic kidney disease; eGFR, estimated glomerular filtration rate.

The changes in eGFR were estimated over a mean ± SD follow-up time of 3.2 ± 1.8 years after baseline using a median (interquartile range) number of eGFR measurements of 9 (4–15). The median (interquartile range) change in eGFR was −1.66 (−2.44 to −0.74) ml/min per 1.73 m^2^ per year in men and −1.50 (−2.15 to −0.63) ml/min per 1.73 m^2^ per year in women. Within both CKD stages 3 and 4, a higher percentage of men than women had a yearly decline in eGFR surpassing 5 ml/min per 1.73 m^2^ and a higher percentage of men had a relatively elevated yearly decline in eGFR between 5 and 2 ml/min per 1.73 m^2^ (Figure 2). The greater decline in eGFR among men compared with women was more pronounced in those starting follow-up in CKD stages 4 and 5 (Figure 2).

During a median (interquartile range) follow-up of 2.57 (1.16–4.08) years in the present analysis, 23% of the men versus 18% of the women initiated KRT (Table 2). In all studied countries with the exception of Brazil, the percentage of patients initiating KRT among men was the same or higher than among women. When KRT was initiated by dialysis, the mean ± SD eGFR was 11.2 ± 4.8 ml/min per 1.73 m^2^ in men versus 10.8 ± 4.7 ml/min per 1.73 m^2^ in women and was slightly higher in men versus women in all countries. This finding was consistent with a lower percentage of late dialysis initiators among men (i.e., with eGFR <5 ml/min per 1.73 m^2^). When KRT was initiated by pre-emptive transplantation, the mean ± SD eGFR was 12.9 ± 5.7 ml/min per 1.73 m^2^ in men versus 13.9 ± 6.8 ml/min per 1.73 m^2^ in women.

**Table 2:** eGFR at KRT initiation and modality of initial KRT by country and sex

Parameter	All		Brazil		France		Germany		US	
	Men	Women	Men	Women	Men	Women	Men	Women	Men	Women
Median follow-up time, yr	2.7 [1.2–4.1]	2.5 [1.1–4.1]	1.5 [0.5–2.0]	1.5 [0.7–2.0]	4.0 [2.8–4.5]	4.0 [3.0–4.7]	2.7 [1.2–4.9]	3.2 [1.6–5.7]	1.3 [0.6–2.0]	1.3 [0.7–2.0]
Patients reaching KRT, *n*	1093	621	50	53	408	210	491	222	167	155
% of cohort	23	18	13	15	21	21	33	20	17	17
eGFR^a^ at dialysis initiation	10.2 [7.9–13.7]	9.9 [7.1–13.3]	12.0 [10.0–20.8]	11.5 [9.0–16.7]	9.2 [7.2–11.9]	8.7 [6.6–11.8]	10.7 [8.4–14.2]	10.3 [7.3–14.8]	10.9 [8.6–14.9]	9.8 [7.1–13.0]
<5	3	5	0	0	5	8	2	4	1	1
5–9.9	45	46	13	28	52	54	43	40	40	51
≥10	53	49	87	72	43	38	56	56	59	48
eGFR^a^ at transplantation^b^	12.0 [9.5–15.0]	12.9 [10.1–16.1]	11.5 [8.0–15.0]	–	12.0 [9.5–14.0]	12.9 [10.5–16.1]	6.6 [4.8–12.2]	–	16.0 [10.7–18.0]	19.7 [6.9–33.5]
<5	3	0	0	–	2	0	33	–	0	0
5–9.9	25	24	50	–	25	20	33	–	22	50
≥10	71	76	50	–	74	80	33	–	78	50
Median time when eGFR was measured before KRT initiation, d	17 [50–5]	21 [61–7]	42 [104–14]	63 [108–40]	15 [36–4]	19 [49–6]	15 [42–6]	15 [47–7]	62 [109–15]	48 [91–16]
Modality of initial KRT										
Dialysis (PD or HD)	93	93	92	89	87	87	99	100	93	92
HD	86	84	91	81	86	84	88	88	79	79
PD	14	16	9	19	14	16	12	12	21	21
Kidney transplant	7	7	8	11	13	13	1	0	7	8

eGFR, estimated glomerular filtration rate; HD, hemodialysis; KRT, kidney replacement therapy; PD, peritoneal dialysis; US, United States.

a The most recent eGFR within 6 mo of KRT initiation; value reported in median [interquartile range], frequency.

b The number of patients who received a transplant was 10 in Brazil, 81 in France, 7 in Germany, and 27 in the US.

The modality of the initial KRT was dialysis in >90% of patients of both sexes (Table 2). Interestingly, France had a markedly higher percentage of patients than other countries in whom the modality of initial KRT was kidney transplantation, rather than dialysis, at an equal rate between men and women. As found in Table 2, pre-emptive transplantation occurred more frequently in men than in women of all 4 countries except France, consistent with our previous report from the European Renal Association-European Dialysis and Transplant Association.^7^ In line with this finding, a higher percentage of men than women were on the transplant waiting list in all 4 countries except France (Table 3). Men seemed to be somewhat less likely than women to initiate dialysis by peritoneal dialysis rather than hemodialysis overall, but this finding was not apparent in the United States. We observed meaningful sex-specific differences in preparation for KRT (Table 3), as a higher percentage of men than women (39% vs. 34%) had a dialysis access created 1 year before their first eGFR being <20 ml/min per 1.73 m^2^. The type of dialysis access that was created was more frequently vascular and less frequently peritoneal in men, in all countries, and vice versa in women.

**Table 3:** Preparation for KRT by country and sex

Parameter	All		Brazil		France		US	
	Men	Women	Men	Women	Men	Women	Men	Women
Patients, *n*	1462	1038	148	136	903	502	411	400
Preparation for KRT^a^								
Access creation	39	34	45	37	44	39	26	27
Type of access^b^								
Vascular access	87	82	94	78	87	86	81	73
Peritoneal access	14	18	6	22	13	14	19	28
On the transplant waiting list	23	22	11	12	28	33	15	12

Restricted to patients with eGFR <20 ml/min per 1.73 m^2^.

eGFR, estimated glomerular filtration rate; KRT, kidney replacement therapy; US, United States.

a Based on interval summary data since 1 yr before the first eGFR < 20 ml/min per 1.73 m^2^; overall 6% missing, 13% missing in Brazil, 0% missing in France, and 4% missing in the US.

b Among patients with data on which type of access was created; overall 24% missing, 40% missing in Brazil, 0% missing in France, and 17% missing in the US.

### Cumulative Incidence and Competing Risks of Dialysis, Transplantation, and Pre-KRT Death

**Figure 3:**
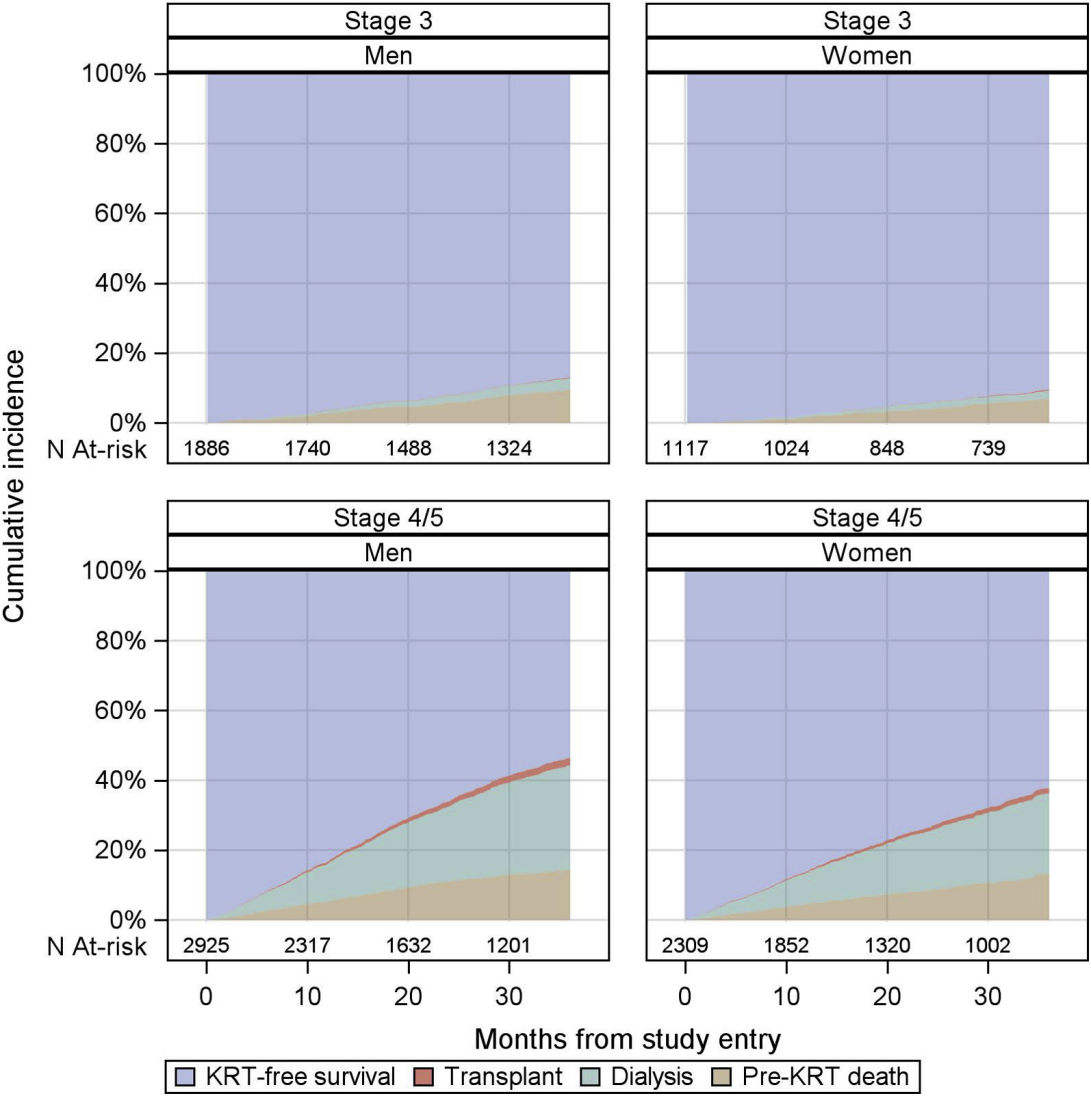
Cumulative incidences of dialysis, transplant, and all-cause deaths in 3 years of follow-up by CKD stage at study entry and sex. CKD, chronic kidney disease; KRT, kidney replacement therapy; N, number.

Sex-specific cumulative incidence curves for dialysis, pre-emptive kidney transplantation, and pre-KRT death are found in Figure 3, and crude adverse event rates, including hospitalizations, are reported in Table 4 (upper). The crude KRT initiation rates (including dialysis and transplantation) were 8.3 per 100 patient years in men and 6.8 per 100 patient years in women. Competing with this event, the crude pre-KRT death rates per 100 patient years were 5.1 in men and 4.2 in women. The results of the Fine and Gray proportional hazards model analysis are found in Table 4 (lower). With time at risk starting from 12 months after study enrollment, the age- and race-adjusted SHRs (95% CIs) of men versus women were 1.51 (1.27–1.80) for dialysis initiation, 1.25 (1.02–1.54) for pre-KRT death, and 1.31 (0.73–2.36) for transplantation (model 2). After additional adjustment for diabetes, cardiovascular disease, and albuminuria, the SHRs were 1.32 (1.10–1.59) for dialysis initiation, 1.14 (0.92–1.40) for pre-KRT death, and 1.44 (0.76–2.74) for transplantation (model 6). After additional adjustment for eGFR slope and first eGFR in the first 12 months after study enrollment, the SHRs were 1.50 (1.25–1.80) for dialysis initiation, 1.15 (0.93–1.42) for pre-KRT death, and 1.53 (0.79–2.94) for transplantation (model 7). Adjustment for eGFR slope did not result in a lower male-to-female SHR for either dialysis or transplantation, in an additional model (model 8).

**Table 4:** Crude adverse event rate in 3 yr of follow-up by sex and eGFR at study entry and Fine and Gray SHRs of men vs. women for dialysis, transplant, and all-cause pre-KRT deaths throughout 3 yr of follow-up

Parameter	Men			Women		
	Overall	Stage 3	Stage 4/5	Overall	Stage 3	Stage 4/5
Patients, *n*	4811	1886	2925	3426	1117	2309
Median follow-up time, yr	2.7	3.0	2.0	2.5	3.0	2.0
Adverse event, n (rate, per 100 patient-yr)						
Hospitalizations^a^	1240 (17.6)	550 (14.4)	690 (21.3)	762 (16.7)	270 (12.7)	492 (20.2)
KRT^b^	842 (8.3)	57 (1.2)	785 (14.2)	483 (6.8)	25 (0.9)	458 (10.3)
Dialysis	785 (7.7)	53 (1.1)	732 (13.2)	451 (6.3)	22 (0.8)	429 (9.7)
Kidney transplantation	61 (0.6)	4 (0.1)	57 (1.0)	33 (0.5)	3 (0.1)	30 (0.7)
Death before KRT	515 (5.1)	156 (3.3)	359 (6.5)	300 (4.2)	61 (2.3)	239 (5.4)
Death^c^	612 (6.0)	162 (3.5)	450 (8.1)	358 (5.0)	64 (2.4)	294 (6.6)

Fine and Gray SHR (95% CI)	Pre-KRT death	Dialysis	Transplant
Model 1	1.20 (0.97–1.47)	1.49 (1.25–1.78)	1.16 (0.65–2.07)
Model 2	1.25 (1.02–1.54)	1.51 (1.27–1.80)	1.31 (0.73–2.36)
Model 3	1.25 (1.02–1.53)	1.50 (1.26–1.78)	1.35 (0.75–2.44)
Model 4	1.15 (0.94–1.42)	1.47 (1.23–1.76)	1.44 (0.78–2.66)
Model 5	1.22 (1.00–1.50)	1.36 (1.14–1.63)	1.25 (0.69–2.27)
Model 6	1.14 (0.92–1.40)	1.32 (1.10–1.59)	1.44 (0.76–2.74)
Model 7	1.15 (0.93–1.42)	1.50 (1.25–1.80)	1.53 (0.79–2.94)
Model 8	1.14 (0.93–1.40)	1.44 (1.20–1.73)	1.41 (0.69–2.86)

CKD, chronic kidney disease; eGFR, estimated glomerular filtration rate; KRT, kidney replacement therapy; SHR, subdistribution hazard ratio.

Models stratified by CKD stage at study entry and accounting for facility clustering.

Model 1: Unadjusted.

Model 2: Adjusted for age, Black race (equals model A [abstract]).

Model 3: Model 2 + diabetes comorbidity.

Model 4: Model 2 + cardiovascular disease (including coronary artery disease, cerebrovascular disease, congestive heart failure, and other cardiovascular diseases).

Model 5: Model 2 + albuminuria.

Model 6: Model 2 + diabetes comorbidity, cardiovascular disease, albuminuria (equals model B [abstract]).

Model 7: Model 6 + first eGFR in first 12 mo after study enrollment as baseline eGFR + eGFR slope in first 12 mo after study enrollment as continuous variable (equals model C [abstract]).

Model 8: Model 6 + last eGFR in first 12 mo after study enrollment as baseline eGFR + eGFR slope in first 12 mo after study enrollment as continuous variable.

a Hospitalization data are not available for Germany.

b Includes dialysis and kidney transplantation.

c Includes death occurred within 1 yr after patient’s departure.

For clarity, unadjusted Kaplan–Meier curves for cumulative probability estimates of KRT in 3 years of follow-up are found in Supplementary Figure S1. Moreover, a sensitivity analysis that excludes individuals who received a pre-emptive transplant is found in Supplementary Table S2.

### Country-Specific Results

Country-specific results are found in Supplementary Tables S3 to S5. Higher central venous catheter use for vascular access at hemodialysis initiation in women (33% vs. 28% in men) was mainly driven by France, whereas women who started hemodialysis in Brazil actually had relatively fewer catheters than men (Supplementary Table S3; data missing for Germany). Regarding pre-KRT death, although the SHRs of men versus women were >1 in all 4 countries, statistical significance was only reached in Brazil, for most of the analyzed models (Supplementary Table S5). Importantly, regarding dialysis initiation, the SHR of men versus women was very close to 1 in the United States, <1 in Brazil, and only slightly >1 in France, without reaching statistical significance in any of these 3 countries and in any of the models (Supplementary Table S5). Overall results were driven by findings from Germany, where the adjusted SHRs for pre-KRT death ranged from 1.82 (1.51–2.20) in model 1 to 1.47 (1.19–1.91) in model 8. Country-specific results for pre-emptive transplantation are only found for France and the United States, as there were too few events in the other countries to yield meaningful results (Table 2). The SHRs of men versus women for pre-emptive transplantation were consistently >1 in these countries and significant in all adjusted models in France but not significant in the United States (Supplementary Table S5).

## Discussion

To the best of our knowledge, we are providing the first international comparison of sex differences in mortality and dialysis incidence of patients with moderate-to-severe CKD followed in nephrologist-led clinics, on top of previous, noncomparative analyses.^20^, ^21^, ^22^ Our most important study finding was that the overall cumulative incidence of KRT, the KRT rate per 100 patient years, and the SHRs for KRT (by dialysis and pre-emptive transplantation) were distinctly higher among men compared with women and were also higher than the respective values for death. However, while the fully adjusted SHR of men versus women for dialysis initiation was 1.44 in all countries, the respective country-specific SHRs were close to 1 in Brazil (SHR = 0.93), France (SHR = 1.02), and the United States (SHR = 1.03), and only slightly >1.44 in Germany (SHR = 1.47 [1.19–1.81]). The US CKDopps results therefore differ from those obtained in a previous analysis of the Chronic Renal Insufficiency Cohort study.^20^ Our CKDopps results from Brazil and France indicate that more country-specific analyses are needed, on top of a recent study from Sweden^22^ and another one from Italy,^21^ and in line with our previous interpretation, that meaningful country differences in the percentages of women receiving dialysis in the Dialysis Outcomes and Practice Patterns Study suggest dialysis initiation does not only depend on different biology between the sexes. Rather, the existence of such country differences renders plausible that dialysis initiation is also subject of gender disparity,^5^ or (more sophisticated) different decision-making according to gender constructs among patients and their care takers.^18^

In an important meta-analysis on 2 million participants (54% women) from general population and high-risk cohorts, conducted by the CKD Prognosis Consortium, Nitsch *et al.*^28^ analyzed the mortality risk for women and men, by eGFR, and found that the slopes of the risk relationship for all-cause mortality and cardiovascular mortality were steeper in women as compared with men. Specifically, compared with an eGFR of 95 ml/min per 1.73 m^2^, the adjusted HR for all-cause mortality at eGFR 45 ml/min per 1.73 m^2^ was 1.32 (95% CI 1.08–1.61) in women and 1.22 (1.00–1.48) in men (*P*_interaction_ < 0.01). The slope being steeper for women led to the authors’ conclusion that “kidney disease is not less important in women than in men,” but they also discussed “competing mortality,” in other words that women, relative to men, become more likely to die at decreasing eGFR levels, despite the fact that their absolute death risk remains lower than that of the men.^28^ With respect to the questions raised by the publication of Nitsch *et al.*,^28^ our study provides an analysis on competing risks of mortality versus KRT initiation in a sex-specific fashion. In contrast to the study of Nitsch *et al.*,^28^ our data were entirely derived from patients with known CKD who were under nephrological care. In our analysis, the risk of dying was higher in men than in women, which is compatible with the study of Nitsch and with the well known, but incompletely understood differences in life expectancy between men and women.^29^, ^30^, ^31^ The fact that men in our study were more often smokers than women and more frequently had cardiovascular disease and cancer might also be in line with their higher death risk. Nevertheless, the chance of receiving KRT in men versus women (SHR = 1.49 [1.25–1.78] in model 7) was higher than the risk of dying (SHR = 1.20 [0.97–1.49] in model 7).

Adjusting for albuminuria markedly reduced the male-versus-female SHR for dialysis (as did adjusting for cardiovascular disease [albeit to a lesser extent]), but adjusting for eGFR slope increased the respective HR. Adjusting for cardiovascular disease attenuated the SHR of men versus women for pre-KRT death, which became statistically insignificant. Although these findings may help explain the preponderance of men in the dialysis population, they might also be indicating that even in the CKD setting, dialysis initiation does not follow a unified, prespecified algorithm among men and women.

Symptoms might be an important factor influencing the decision to initiate KRT. Nevertheless, a previous analysis from the European QUALity Study on the treatment of advanced CKD revealed that women reported more CKD-related symptoms than men.^32^ This important observation may not be well known, although being consistent with the finding that women with CKD more often than men reported only fair or poor health and that their health-related quality of life was lower than the men’s in all dimensions, especially in the symptoms and burden dimensions.^33^ These findings, together, are also counterarguments against biological reasons (symptom burden) explaining the under-representation of women in the dialysis population. Nevertheless, the present analysis did not actually analyze such factors in the CKDopps data set, such that the interpretation remains speculative.

We observed faster CKD progression in men when compared with women, in agreement with a recent review of the literature,^4^ which put an end to previous controversy.^34^^,^^35^ The opposite side of the coin, which are slower CKD progression among women and fewer CKD-related symptoms among women versus men, were the principal arguments in favor of the hypothesis that women “do not have to go” to dialysis. Nevertheless, one of our perhaps most important study findings was that adjustment for eGFR slope did not result in a meaningfully lower SHR of men versus women for either dialysis or transplantation in 2 distinct models (assuring the robustness of the calculation). This finding indicated that slower progression of kidney disease in women cannot explain the higher chance of men to receive KRT because if faster progression among men had resulted in a higher SHR of men versus women for dialysis, this SHR would have been expected to decline after adjustments. Although adjusting for additional biological differences attenuated the SHR of men versus women for dialysis (especially adjusting for albuminuria, besides cardiovascular disease and diabetes [models 3–6 in Table 4]), a statistically significant and clinically meaningful difference between men and women prevailed.

Although the international design of CKDopps and thus of the present analysis is a study strength, some of the results are not novel. For example, Stengel *et al.*^33^ have previously reported in their analysis of CKD-Renal Epidemiology and Information Network patients (partly overlapping with the CKDopps patients from France in the present study) that men predominate in the CKD-Renal Epidemiology and Information Network and several other CKD cohorts from the United States,^36^ Japan,^37^ Canada,^38^ Germany,^39^ and the People’s Republic of China.^40^ This finding nevertheless is still surprising, given the higher prevalence of CKD among women,^2^^,^^41^, ^42^, ^43^ and might indicate that the referral practice from the general population onto CKD care is different for men versus women. An additional explanation why the proportion of men in CKDopps clinics was higher than expected might be the large proportion of patients with CKD stage 4+, which reveals that the cohort is not representing the general CKD population (consisting mainly of CKD stage 3^44^). Taking this point further, the question might be raised when during the course of CKD the patients were recruited for CKD care in CKDopps clinics and how this process could have influence the rate of KRT. The present analysis is of course not suited to provide a meaningful answer to this question.

The fact that women had lower eGFR at KRT initiation and more women than men were late dialysis initiators is also consistent with several previous analyses,^45^, ^46^, ^47^, ^48^ all of which were, however, conducted with US patients. A previous analysis suggested some overestimation of CKD among women when eGFR was calculated by the Modification of Renal Diet in Renal Disease formula (−3.1 ± 17.2 ml/min per 1.73 m^2^ compared with chromium ethylenediaminetetraacetic acid clearance for women aged <65 years^49^). The CKD Epidemiology Collaboration formula was, however, used in the present analysis, which has been found to result in a more accurate categorization of mortality risk than the Modification of Renal Diet in Renal Disease formula.^50^ Finally (as stated previously), sex-specific analyses of death and dialysis initiation have been published for the Chronic Renal Insufficiency Cohort study participants,^20^ for an Italian^21^ and a Swedish cohort,^22^ which, however, renders our comparative study more meaningful rather than less novel.

Among additional study limitations, we recognize it cannot be fully ruled out that the inclusion of CKDopps participants from the respective CKD clinics could have introduced an element of bias. When using Fine and Gray models, the magnitude of the association cannot be quantified (but only the direction of the association), and we acknowledge that which model most adequately allows the assessment of competing risks is a matter of debate.^51^ Importantly, the present study could not differentiate between sex and gender. Strictly speaking, sex was by design of the CKDopps questionnaires, an assigned variable, and the fact that we used the terminology “men” and “women” throughout (rather than males and females) is an additional shortcoming, which was accepted to remain consistent with previous work.^5^^,^^7^ Future studies are formally needed to confirm that sex-specific differences in KRT initiation are the same or very similar to differences in KRT initiation, by self-assigned sex and self-assigned gender.

The sex-specific differences observed in the present CKDopps study cannot ascertain or make assumptions on any occurrences outside of the CKD care setting. The “fate” of an individual with CKD, however, may differ, by the timeliness of this individual’s referral to nephrologist care,^52^ and the referral to the nephrologist first depends on the recognition that the patient even has CKD. Our recent results from the National Health and Nutrition Examination Survey^15^ reveal that women less frequently than men report they have been informed of their kidney disease. The lack of CKD awareness among women may explain why those women who were actually included in CKDopps had more advanced kidney disease than men and lower CKD vintage. These and other explanations for the observed findings currently also still remain speculative, as causality cannot formally be established in the setting of an observational study.

In summary, the present study reveals that women with CKD followed in the national sample of clinics led by nephrologists had a lower chance of initiating dialysis, compared with men, with meaningful differences identified by country. Of note, we were unable to identify the factors explaining the differential KRT chance between men and women, as all the available variables adjusted for in the analysis did not explain this difference. If kidney failure requiring therapy develops, the decision to initiate KRT is almost always individual, and not guideline driven.^53^ Awareness that treatment of women may differ from the treatment of men is important for the nephrological community and should be openly discussed and further investigated.

## Disclosure

The results presented in this paper have not been published previously in whole or part, except in Abstract form (American Society of Nephrology, October 22, 2020, Abstract PO0539). This project was carried out as part of the Dialysis Outcomes and Practice Patterns Study program that receives global support from a large consortium of funders listed at https://www.dopps.org/AboutUs/Support.aspx. All such grants were made to Arbor Research Collaborative for Health and not to the coauthors directly. Additional funding to support the CKD-REIN study providing French data has been received from AstraZeneca, Fresenius Medical Care, GlaxoSmithKline, Vifor Fresenius, and Amgen. The authors acknowledge support from the Austrian Science Fund (grant no. KL754-B). CC reports receiving grants from Travere and the Fresenius Medical Care. All the other authors declared no competing interests.

## Supplementary Material

Supplementary File (PDF)

**Figure S1.** Unadjusted Kaplan–Meier curves for cumulative probability estimates of KRT in 3 years of follow-up, by CKD stage at study entry and sex.

**Table S1.** Percentage of women in the CKDopps versus NHANES (2013–2018) by age group and CKD stage.

**Table S2.** Fine-Gray Subdistribution Hazard Ratios (SHRs) of men versus women for dialysis, and all-caused pre-KRT deaths throughout 3 years of follow-up, excluding individuals receiving a pre-emptive transplant.

**Table S3.** Types of vascular access used for initial HD dialysis, by country and sex.

**Table S4.** Crude adverse event rates, by country and sex.

**Table S5.** Fine-Gray Subdistribution Hazard Ratios (SHRs) of men versus women for dialysis, transplant, and all-caused pre-KRT deaths throughout 3 years of follow-up.

## References

[1] Jager K.J., Kovesdy C., Langham R., Rosenberg M., Jha V., Zoccali C. (2019). A single number for advocacy and communication-worldwide more than 850 million individuals have kidney diseases. Kidney Int.

[2] Carrero J.J., Hecking M., Chesnaye N.C., Jager K.J. (2018). Sex and gender disparities in the epidemiology and outcomes of chronic kidney disease. Nat Rev Nephrol.

[3] Murphy D., McCulloch C.E., Lin F. (2016). Trends in prevalence of chronic kidney disease in the United States. Ann Intern Med.

[4] Neugarten J., Golestaneh L. (2019). Influence of sex on the progression of chronic kidney disease. Mayo Clin Proc.

[5] Hecking M., Bieber B.A., Ethier J. (2014). Sex-specific differences in hemodialysis prevalence and practices and the male-to-female mortality rate: the Dialysis Outcomes and Practice Patterns Study (DOPPS). PLoS Med.

[6] United States Renal Data System (2015). https://www.usrds.org/media/2293/vol2_usrds_esrd_15.pdf.

[7] Antlanger M., Noordzij M., van de Luijtgaarden M. (2019). Sex differences in kidney replacement therapy initiation and maintenance. Clin J Am Soc Nephrol.

[8] Kainz A., Berner C., Ristl R. (2019). Sex-specific analysis of haemodialysis prevalence, practices and mortality over time: the Austrian Dialysis Registry from 1965 to 2014. Nephrol Dial Transplant.

[9] Kjellstrand C.M., Logan G.M. (1987). Racial, sexual and age inequalities in chronic dialysis. Nephron.

[10] (2011). Taking sex into account in medicine. Lancet.

[11] Kim A.M., Tingen C.M., Woodruff T.K. (2010). Sex bias in trials and treatment must end. Nature.

[12] (2010). Putting gender on the agenda. Nature.

[13] Bairey Merz C.N., Dember L.M., Ingelfinger J.R. (2019). Sex and the kidneys: current understanding and research opportunities. Nat Rev Nephrol.

[14] Coresh J., Byrd-Holt D., Astor B.C. (2005). Chronic kidney disease awareness, prevalence, and trends among U.S. adults, 1999 to 2000. J Am Soc Nephrol.

[15] Hödlmoser S., Winkelmayer W.C., Zee J. (2020). Sex differences in chronic kidney disease awareness among US adults, 1999 to 2018. PLoS One.

[16] Chandna S.M., Carpenter L., Da Silva-Gane M., Warwicker P., Greenwood R.N., Farrington K. (2016). Rate of decline of kidney function, modality choice, and survival in elderly patients with advanced kidney disease. Nephron.

[17] Morton R.L., Turner R.M., Howard K., Snelling P., Webster A.C. (2012). Patients who plan for conservative care rather than dialysis: a national observational study in Australia. Am J Kidney Dis.

[18] Mauvais-Jarvis F., Bairey Merz N., Barnes P.J. (2020). Sex and gender: modifiers of health, disease, and medicine. Lancet.

[19] Plantinga L.C., Boulware L.E., Coresh J. (2008). Patient awareness of chronic kidney disease: trends and predictors. Arch Intern Med.

[20] Ricardo A.C., Yang W., Sha D. (2019). Sex-related disparities in CKD progression. J Am Soc Nephrol.

[21] Minutolo R., Gabbai F.B., Chiodini P. (2020). Sex differences in the progression of CKD among older patients: pooled analysis of 4 cohort studies. Am J Kidney Dis.

[22] Swartling O., Rydell H., Stendahl M., Segelmark M., Lagerros Y.T., Evans M. (2021). CKD progression and mortality among men and women: a nationwide study in Sweden. Am J Kidney Dis.

[23] Mariani L., Stengel B., Combe C. (2016). The CKD Outcomes and Practice Patterns Study (CKDopps): rationale and methods. Am J Kidney Dis.

[24] Pecoits-Filho R., Fliser D., Tu C. (2019). Prescription of renin-angiotensin-aldosterone system inhibitors (RAASi) and its determinants in patients with advanced CKD under nephrologist care. J Clin Hypertens (Greenwich).

[25] Ahmed S.B., Saad N., Dumanski S.M. (2020). Gender and CKD: beyond the binary. Clin J Am Soc Nephrol.

[26] Levey A.S., Stevens L.A., Schmid C.H. (2009). A new equation to estimate glomerular filtration rate. Ann Intern Med.

[27] Fine J.P., Gray R.J. (1999). A proportional hazards model for the subdistribution of a competing risk. J Am Stat Assoc.

[28] Nitsch D., Grams M., Sang Y. (2013). Associations of estimated glomerular filtration rate and albuminuria with mortality and renal failure by sex: a meta-analysis. BMJ.

[29] Luy M., Gast K. (2014). Do women live longer or do men die earlier? Reflections on the causes of sex differences in life expectancy. Gerontology.

[30] Luy M. (2009). Unnatural deaths among nuns and monks: is there a biological force behind male external cause mortality?. J Biosoc Sci.

[31] Luy M., Di Giulio P., Di Lego V., Lazarevič P., Sauerberg M. (2020). Life expectancy: frequently used, but hardly understood. Gerontology.

[32] van de Luijtgaarden M.W.M., Caskey F.J., Wanner C. (2019). Uraemic symptom burden and clinical condition in women and men of ≥ 65 years of age with advanced chronic kidney disease: results from the EQUAL study. Nephrol Dial Transplant.

[33] Stengel B., Metzger M., Combe C. (2019). Risk profile, quality of life and care of patients with moderate and advanced CKD: the French CKD-REIN Cohort Study. Nephrol Dial Transplant.

[34] Jafar T.H., Schmid C.H., Stark P.C. (2003). The rate of progression of renal disease may not be slower in women compared with men: a patient-level meta-analysis. Nephrol Dial Transplant.

[35] Neugarten J., Acharya A., Silbiger S.R. (2000). Effect of gender on the progression of nondiabetic renal disease: a meta-analysis. J Am Soc Nephrol.

[36] Lash J.P., Go A.S., Appel L.J. (2009). Chronic Renal Insufficiency Cohort (CRIC) study: baseline characteristics and associations with kidney function. Clin J Am Soc Nephrol.

[37] Imai E., Matsuo S., Makino H. (2010). Chronic Kidney Disease Japan Cohort study: baseline characteristics and factors associated with causative diseases and renal function. Clin Exp Nephrol.

[38] Levin A., Rigatto C., Brendan B. (2013). Cohort profile: Canadian study of prediction of death, dialysis and interim cardiovascular events (CanPREDDICT). BMC Nephrol.

[39] Titze S., Schmid M., Köttgen A. (2015). Disease burden and risk profile in referred patients with moderate chronic kidney disease: composition of the German Chronic Kidney Disease (GCKD) cohort. Nephrol Dial Transplant.

[40] Yuan J., Zou X.R., Han S.P. (2017). Prevalence and risk factors for cardiovascular disease among chronic kidney disease patients: results from the Chinese cohort study of chronic kidney disease (C-STRIDE). BMC Nephrol.

[41] Hill N.R., Fatoba S.T., Oke J.L. (2016). Global prevalence of chronic kidney disease—a systematic review and meta-analysis. PLoS One.

[42] Mills K.T., Xu Y., Zhang W. (2015). A systematic analysis of worldwide population-based data on the global burden of chronic kidney disease in 2010. Kidney Int.

[43] Zhang Q.L., Rothenbacher D. (2008). Prevalence of chronic kidney disease in population-based studies: systematic review. BMC Public Health.

[44] Vestergaard S.V., Christiansen C.F., Thomsen R.W., Birn H., Heide-Jørgensen U. (2021). Identification of patients with CKD in medical databases: a comparison of different algorithms. Clin J Am Soc Nephrol.

[45] Crews D.C., Scialla J.J., Liu J. (2014). Predialysis health, dialysis timing, and outcomes among older United States adults. J Am Soc Nephrol.

[46] Slinin Y., Guo H., Li S. (2014). Provider and care characteristics associated with timing of dialysis initiation. Clin J Am Soc Nephrol.

[47] Kausz A.T., Obrador G.T., Arora P., Ruthazer R., Levey A.S., Pereira B.J.G. (2000). Late initiation of dialysis among women and ethnic minorities in the United States. J Am Soc Nephrol.

[48] Li Y., Kapke A., Jin Y. (2013). SA-PO516 estimated GFR at dialysis initiation: associations with clinical and non-clinical factors. J Am Soc Nephrol.

[49] Froissart M., Rossert J., Jacquot C., Paillard M., Houillier P. (2005). Predictive performance of the modification of diet in renal disease and Cockcroft-Gault equations for estimating renal function. J Am Soc Nephrol.

[50] Matsushita K., Mahmoodi B.K., Woodward M. (2012). Comparison of risk prediction using the CKD-EPI equation and the MDRD study equation for estimated glomerular filtration rate. JAMA.

[51] Noordzij M., Leffondré K., van Stralen K.J., Zoccali C., Dekker F.W., Jager K.J. (2013). When do we need competing risks methods for survival analysis in nephrology?. Nephrol Dial Transplant.

[52] Luxton G., CARI (2010). The CARI guidelines. Timing of referral of chronic kidney disease patients to nephrology services (adult). Nephrology (Carlton).

[53] Joly D., Anglicheau D., Alberti C. (2003). Octogenarians reaching end-stage renal disease: cohort study of decision-making and clinical outcomes. J Am Soc Nephrol.

