# Supplementary material for: Sex-Specific Differences in Mortality and Incident Dialysis in the Chronic Kidney Disease Outcomes and Practice Patterns Study
Source: Kidney Int Rep. 2021 Dec 1;7(3):410–23. doi: 10.1016/j.ekir.2021.11.018 (PMC8897674; doi:10.1016/j.ekir.2021.11.018)
Supplement: Supplementary File (PDF) [file mmc1.pdf]

Supplemental Figure 1. Unadjusted Kaplan-Meier curves for cumulative probability estimates of KRT in 3 years of follow-up, by CKD stage at study entry and sex.

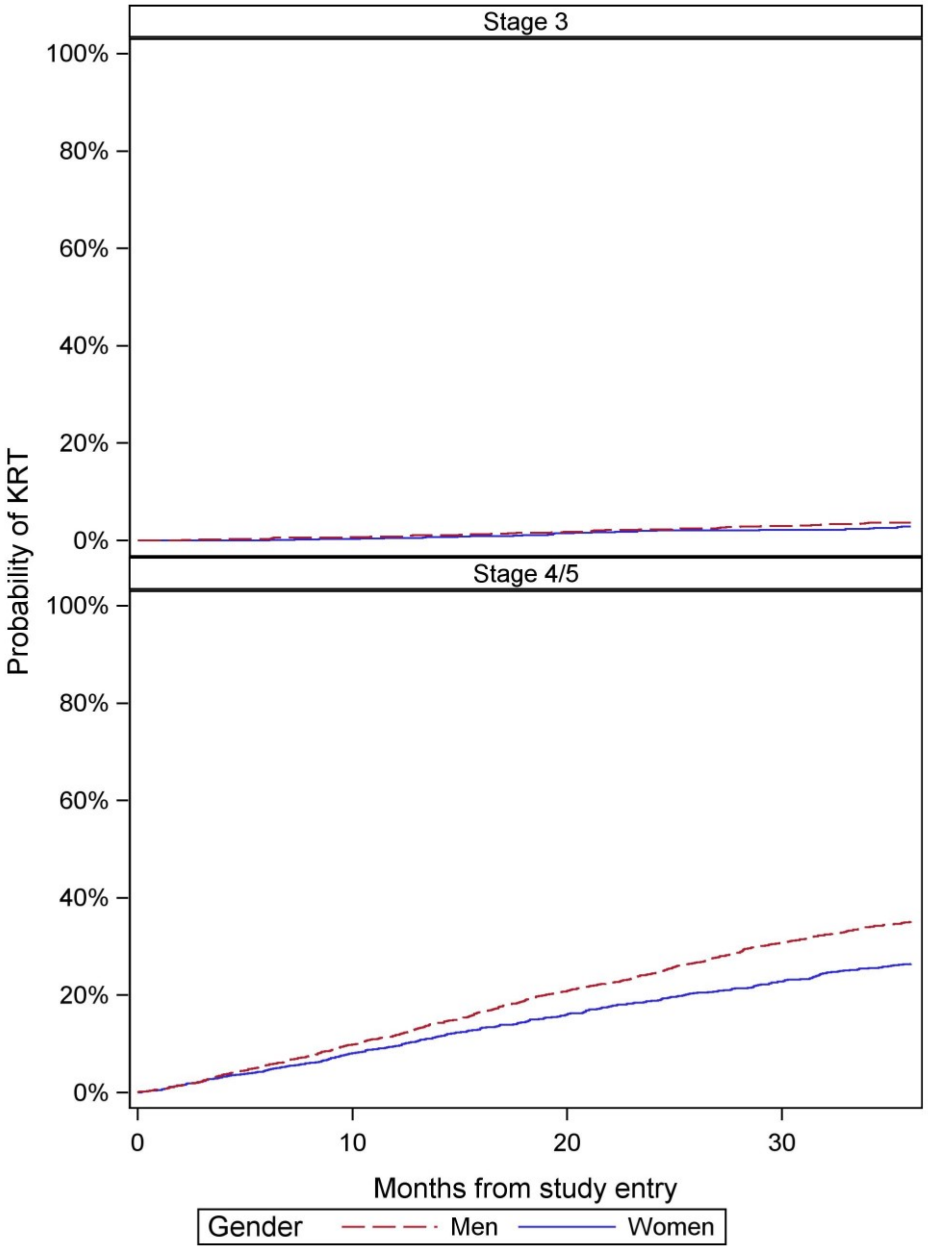

Supplemental Tables

Supplemental Table 1. Percentage of women in the CKDopps versus NHANES (2013-2018) by age group and CKD stage.

Age group	CKD stage	CKDOPPS				NHANES (95% CI)
		Brazil	France	Germany	US	
18-44	3	57	50	38	45	39.4 (17.1, 61.6)
	4/5	58	44	40	50	57.7 (26.1, 89.4)
45-54	3	41	34	33	51	55.7 (40.9, 70.6)
	4/5	45	51	39	52	54.0 (21.4, 86.5)
55-64	3	50	35	40	44	48.8 (37.6, 60.1)
	4/5	50	43	40	50	39.5 (19.9, 59.1)
65-74	3	35	28	37	46	57.0 (49.4, 64.5)
	4/5	48	34	39	49	58.0 (41.4, 74.6)
75+	3	39	32	40	45	59.4 (54.8, 64.1)
	4/5	50	32	49	50	70.2 (57.1, 83.2)

Note: CKD stage is classified based on eGFR using CKD-EPI formula

Supplemental Table 2. Fine-Gray Subdistribution Hazard Ratios (SHRs) of men versus women for dialysis, and all-caused pre-KRT deaths throughout 3 years of follow-up, excluding individuals receiving a pre-emptive transplant.

Fine-Gray SHR (95% CI)	Pre-KRT death	Dialysis
Model 1	1.27 (1.07, 1.51)	1.40 (1.24, 1.59)
Model 2	1.30 (1.10, 1.54)	1.40 (1.24, 1.58)
Model 3	1.30 (1.10, 1.53)	1.40 (1.24, 1.57)
Model 4	1.22 (1.03, 1.45)	1.38 (1.22, 1.56)
Model 5	1.22 (1.03, 1.45)	1.19 (1.05, 1.35)
Model 6	1.15 (0.97, 1.36)	1.18 (1.05, 1.33)
Model 7	1.16 (0.98, 1.37)	1.27 (1.11, 1.44)
Model 8	1.15 (0.97, 1.37)	1.23 (1.07, 1.40)

Models stratified by CKD stage at study entry and accounting for facility clustering.
Model 1: Unadjusted
Model 2: Adjusted for age, Black race (equals model A [Abstract])
Model 3: Model 2 + diabetes comorbidity
Model 4: Model 2 + cardiovascular disease (including coronary artery disease, cerebrovascular disease, congestive heart failure, and other cardiovascular disease)
Model 5: Model 2 + albuminuria
Model 6: Model 2 + diabetes comorbidity, cardiovascular disease, albuminuria (equals model B [Abstract])
Model 7: Model 6 + first eGFR in first 12 months after study enrolment as baseline eGFR + eGFR slope in first 12 months after study enrolment as continuous variable (equals model C [Abstract])
Model 8: Model 6 + last eGFR in first 12 months after study enrolment as baseline eGFR + eGFR slope in first 12 months after study enrolment as continuous variable

Supplemental Table 3. Types of vascular access used for initial HD dialysis, by country and sex.

	All		Brazil		France		US	
	Men	Women	Men	Women	Men	Women	Men	Women
Patients, N	341	188	30	18	269	133	42	37
Central venous catheter	28%	33%	27%	17%	26%	33%	41%	41%
Arteriovenous fistula	70%	61%	73%	83%	72%	64%	57%	38%
Arteriovenous graft	2%	6%	0%	0%	2%	3%	2%	22%

Supplemental Table 4. Crude adverse event rates, by country and sex.

	All		Brazil		France		Germany		US	
	Men	Women	Men	Women	Men	Women	Men	Women	Men	Women
Patients, N	4929	3544	463	417	1944	1024	1479	1109	1043	994
Median follow-up time, years	2.7	2.5	1.8	1.7	4.0	4.0	2.6	3.1	1.2	1.3
**Adverse event rate, per 100 patient-years**										
Hospitalizations[a]	18.6	18.2	17.9	13.4	17.5	15.9	-	-	25.3	27.6
KRT[b]	8.3	6.7	9.4	10.6	6.0	5.7	10.7	5.5	11.0	9.8
Death before KRT	4.9	4.2	3.2	2.1	4.1	3.0	5.7	4.9	7.4	6.1
Death[c]	7.0	5.4	5.0	3.0	4.6	3.7	10.6	7.2	8.8	6.7

[a] Hospitalization data are not available for Germany

[b] Includes dialysis and kidney transplantation

[c] Includes death after ESRD; follow-up time for death after ESRD is ended at 12 months after patient's departure from the study

Supplemental Table 5. Fine-Gray Subdistribution Hazard Ratios (SHRs) of men versus women for dialysis, transplant, and all-caused pre-KRT deaths throughout 3 years of follow-up.

	Brazil			France			Germany			US		
Fine–Gray SHR (95% CI)	Pre-KRT death	Dialysis	Transplant	Pre-KRT death	Dialysis	Transplant	Pre-KRT death	Dialysis	Transplant	Pre-KRT death	Dialysis	Transplant
Model 1	2.04 (0.99,4.19)	0.98 (0.70,1.37)	-	1.45 (1.05,2.00)	1.13 (0.89,1.44)	1.43 (0.78,2.63)	1.04 (0.78,1.38)	1.91 (1.57,2.32)	-	1.21 (0.93,1.57)	0.98 (0.80,1.20)	1.21 (0.48,3.01)
Model 2	2.15 (1.05,4.42)	0.97 (0.68,1.4)	-	1.33 (0.96,1.83)	1.16 (0.91,1.48)	1.89 (1.13,3.15)	1.18 (0.88,1.58)	1.82 (1.51,2.20)	-	1.22 (0.95,1.56)	1.02 (0.82,1.27)	1.32 (0.55,3.15)
Model 3	2.16 (1.08,4.33)	0.98 (0.68,1.40)	-	1.30 (0.95,1.79)	1.16 (0.91,1.47)	1.93 (1.17,3.18)	1.18 (0.88,1.57)	1.78 (1.48,2.13)	-	1.21 (0.95,1.55)	1.03 (0.83,1.28)	1.31 (0.55,3.13)
Model 4	2.13 (1.05,4.30)	0.97 (0.67,1.39)	-	1.18 (0.86,1.62)	1.14 (0.90,1.44)	2.01 (1.22,3.32)	1.15 (0.85,1.54)	1.79 (1.48,2.16)	-	1.13 (0.87,1.45)	1.02 (0.83,1.27)	1.36 (0.56,3.32)
Model 5	2.01 (0.98,4.13)	0.94 (0.66,1.34)	-	1.22 (0.89,1.68)	0.99 (0.78,1.27)	1.82 (1.10,3.02)	1.16 (0.87,1.55)	1.58 (1.29,1.93)	-	1.19 (0.92,1.54)	0.97 (0.77,1.21)	1.26 (0.52,3.03)
Model 6	2.00 (1.00,4.02)	0.94 (0.65,1.35)	-	1.10 (0.81,1.50)	0.98 (0.77,1.25)	1.96 (1.21,3.17)	1.13 (0.84,1.52)	1.52 (1.25,1.86)	-	1.11 (0.86,1.44)	0.99 (0.79,1.23)	1.28 (0.52,3.13)
Model 7	2.01 (1.00,4.05)	0.94 (0.65,1.35)	-	1.11 (0.81,1.50)	1.07 (0.84,1.37)	1.95 (1.17,3.27)	1.14 (0.85,1.54)	1.55 (1.27,1.90)	-	1.11 (0.86,1.44)	1.05 (0.83,1.33)	1.36 (0.55,3.35)
Model 8	2.01 (1.00,4.05)	0.93 (0.64,1.35)	-	1.09 (0.80,1.48)	1.02 (0.79,1.31)	1.83 (1.03,3.24)	1.13 (0.84,1.51)	1.47 (1.19,1.81)	-	1.12 (0.87,1.45)	1.03 (0.81,1.31)	1.33 (0.54,3.27)

Note: The results of Transplant models for Brazil and Germany are not shown as there were too few outcomes
Models stratified by CKD stage at study entry and accounting for facility clustering.
Model 1: Unadjusted
Model 2: Adjusted for age, Black race (equals model A [Abstract])
Model 3: Model 2 + diabetes comorbidity
Model 4: Model 2 + cardiovascular disease (including coronary artery disease, cerebrovascular disease, congestive heart failure, and other cardiovascular disease)
Model 5: Model 2 + albuminuria
Model 6: Model 2 + diabetes comorbidity, cardiovascular disease, albuminuria (equals model B [Abstract])
Model 7: Model 6 + first eGFR in first 12 months after study enrolment as baseline eGFR + eGFR slope in first 12 months after study enrolment as continuous variable (equals model C [Abstract])
Model 8: Model 6 + last eGFR in first 12 months after study enrolment as baseline eGFR + eGFR slope in first 12 months after study enrolment as continuous variable
